# Shallow Inferior Conjunctival Fornix in Contracted Socket and Anophthalmic Socket Syndrome: A Novel Technique to Deepen the Fornix Using Fascia Lata Strips

**DOI:** 10.1155/2016/3857579

**Published:** 2016-05-30

**Authors:** Mohamed F. K. Ibrahiem, Sahar T. A. Abdelaziz

**Affiliations:** Oculoplastic Unit, Ophthalmology Department, El Minia University Hospital, 98 Korneesh El Nile Street, Minia 61111, Egypt

## Abstract

*Purpose.* To evaluate a new surgical technique using fascia lata to deepen the shallow inferior conjunctival fornix in contracted socket and anophthalmic socket syndrome.* Methods.* A prospective controlled study in which 24 sockets of 24 patients who were unable to wear and retain their ocular prosthesis due to shallow inferior fornix were enrolled and categorized into anophthalmic socket syndromes (9 patients) and contracted sockets (15 patients). Another 24 patients who underwent evisceration or enucleation with healthy sockets and can wear and retain their prosthesis comfortably were chosen as a control group. Deepening of the fornix was performed using fascia lata strips under general anesthesia. Central depth of the inferior fornix was measured preoperatively and postoperatively.* Results. *A statistically significant improvement of the postoperative central inferior fornix depth was reported which was marked in anophthalmic subgroup. 100% of anophthalmic sockets and 93.3% of contracted sockets achieved satisfactory results during the follow-up period with no postoperative lower eyelid malposition or obvious skin scar.* Conclusion. *Fascia lata technique is a new alternative and effective procedure to deepen the shallow inferior fornix that can be used in moderate to severe contracted sockets or anophthalmic socket syndrome with minimal lower eyelid or socket complications.

## 1. Introduction

Adequate retention of the ocular prosthesis in the anophthalmic socket requires a well-formed inferior fornix, which in turn requires sufficient conjunctival length and a deep recess. Obliteration of the inferior fornix might occur in contracted socket and in anophthalmic socket (postenucleation socket) syndrome [1].

Contracted socket is a condition characterized by fibrosis of the anophthalmic socket where shallow or obliterated fornix is a key finding in different stages of the disease and it occurs secondary to conjunctival shrinkage [2].

Anophthalmic socket syndrome encompasses several anomalies including shallow lower fornix. The later occurs possibly as there is no globe and so the inferior rectus muscle is at a higher level in the socket with subsequent elevation of the lower lid retractors and their connections including the fornical conjunctiva [3].

Management of the shallow inferior fornix in contracted socket is usually done by deepening sutures following mucous membrane graft to avoid lower lid entropion [3].

In anophthalmic socket syndrome, the shallow inferior fornix is reformed by deepening sutures that exit at the skin of the lower lid where they are tied over bolsters and left for 2-3 weeks [3]. However skin erosion and infection necessitated early removal of the externalized sutures and increased the risk of recurrence [4].

A transconjunctival inferior fornix fixation is another method of repair of shallow inferior fornix in anophthalmic socket syndrome where the edges of the conjunctival incision are directly sutured to the periosteum with no need for externalized sutures and stents [5].

Although this procedure yielded good results, it still carries the risk of triggering contracture of the socket as it involved dissection of the fornical conjunctiva and lower lid retractors with cauterization of the tissues and excessive sacrifice or destruction of conjunctiva [6].

For the above reason, we designed this study to evaluate new surgical technique for deepening the inferior conjunctival fornix using fascia lata in contracted socket and anophthalmic socket syndrome.

## 2. Patients and Methods

### 2.1. Design

This was a prospective, nonrandomized interventional case series studythat was carried out in the Oculoplastic Unit at Ophthalmology Department, El Minia University Hospital, in the period from February 2009 to May 2012.

All procedures performed in studies involving human participants were in accordance with the ethical standards of the institutional and/or national research committee and with the 1964 Helsinki declaration and its later amendments or comparable ethical standards.

### 2.2. Patient Selection

All patients attending to the Oculoplastic Unit at El Minia University Hospital who were unable to wear and retain their ocular prosthesis due to shallow or obliterated inferior conjunctival fornix were examined and 24 male and female adult patients were considered for study enrollment after exclusion of patients with previous surgical correction of shallow inferior conjunctival fornix.

The patient's sockets were examined thoroughly and the patients were categorized into 2 subgroups according to the cause of the shallow inferior fornix as follows:

1. Anophthalmic group: 9 patients with anophthalmic socket syndrome.
2. Contracted group: 15 patients with moderate to severe contracted sockets.

Another 24 patients who underwent evisceration or enucleation with healthy sockets and can wear and retain their prosthesis comfortably were chosen as a control group.

Indication for evisceration or enucleation and time elapsed since that surgery were recorded for every patient in the control as well as patient groups.

The depth of the inferior conjunctival fornix was measured in mm at the center of the lower eyelid in all participants in the control group and in patient subgroups.

Informed consents were obtained from all patients for the surgery

### 2.3. Surgical Technique 

#### 2.3.1. Fascia Lata Harvesting

Under general anesthesia and after sterilization of the thigh with povidone iodine, a 2 cm vertical skin incision was made 10 cm above the lateral epicondyle of the knee over the iliotibial tract and then blunt dissection of the tissues was carried out down to the fascia lata site.

A 3 mm wide free end of the fascia was fashioned using scissors and then the Masson fascia lata stripper was engaged and advanced for 15 cm upwards towards the hip joint and then triggered several times till a fascia lata strip 3 mm wide and 10–15 cm in length was obtained.

#### 2.3.2. Reformation of the Inferior Fornix

**Figure 1:**
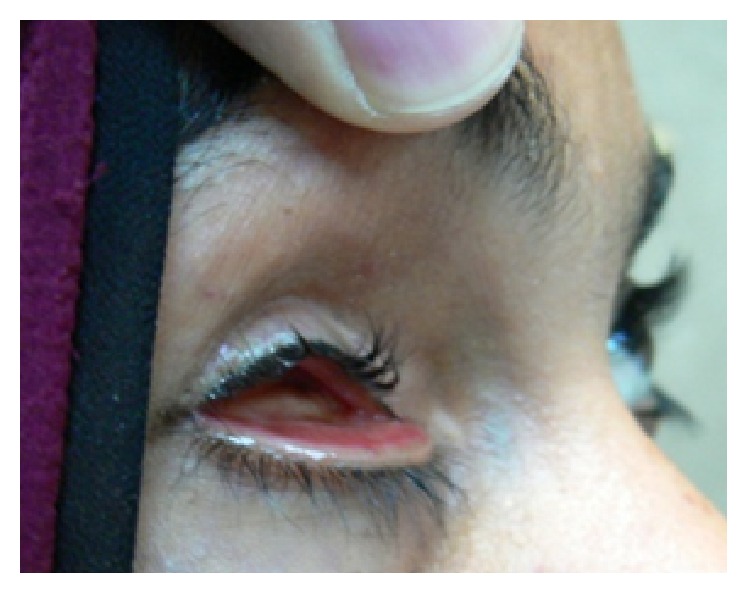
Patient with anophthalmic socket syndrome having shallow inferior fornix and lax lower eyelid.

*(1) In Case of Anophthalmic Socket with Abundant Conjunctiva but Shallow Inferior Fornix (Figure 1)*. A 2 mm conjunctival incision was made in each side of the center of the inferior fornix.

Another three horizontal cutaneous incisions were made in the lower lid over the inferior orbital margin, each of which was 5 mm in length and deepened down to the periosteum.

**Figure 2:**
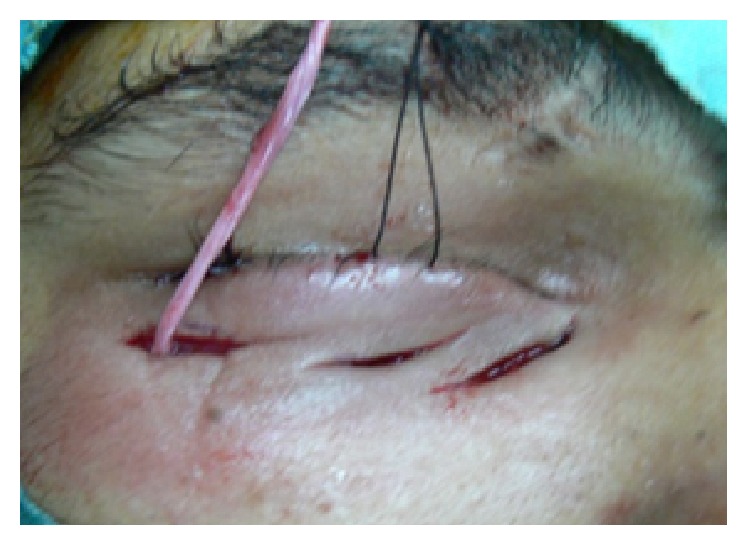
Intraoperatively 3 deep skin incisions were made with one end of the fascia strip secured to the periosteum of the inferior orbital margin.

The lateral incision was at the level of lateral canthus, the medial one was at the level of the lower punctum, and the middle one was in between (Figure 2).

**Figure 3:**
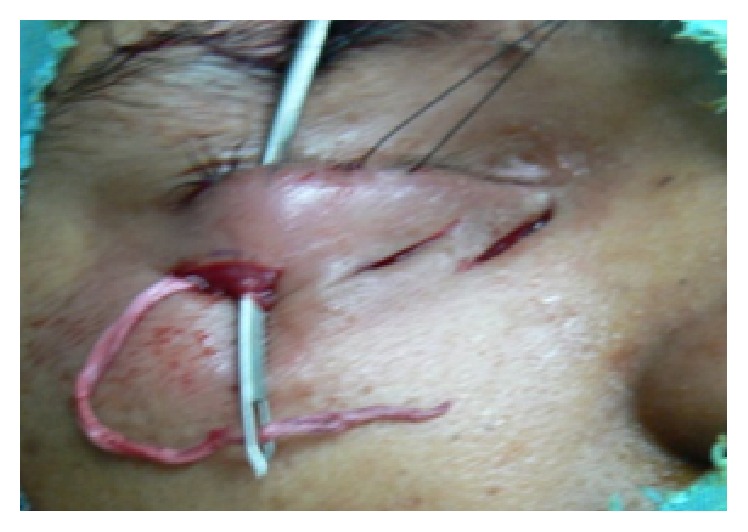
Intraoperatively Wright's fascia needle was used to pass the free end of the fascia strip from skin to conjunctival incision.

*In the Lateral Cutaneous Incision*. One end of the fascia lata strip was secured to the periosteum using a nonabsorbable polyester 5/0 suture (Figure 2) and then Wright's fascia needle was used to deliver the free end of the fascia strip to lateral conjunctival incision (Figure 3) and then to the middle cutaneous incision.

*In the Middle Cutaneous Incision*. The free end of the fascia lata strip was passed under periosteal tunnel created to act as a pulley to change direction of pull and so facilitate intraoperative adjustment.

Again Wright's needle was used to pass the free end of the fascia strip to the medial conjunctival incision then and to the medial cutaneous incision.

**Figure 4:**
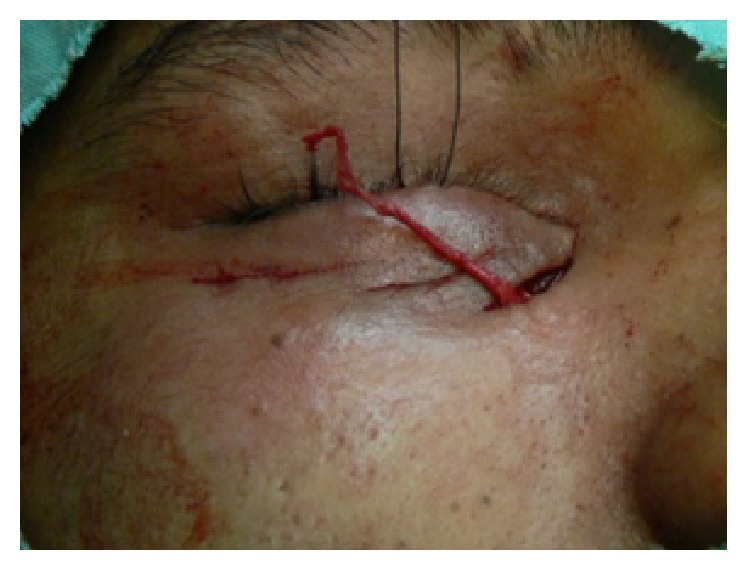
Intraoperatively fascia strip emerged through the periosteal tunnel in the medial skin wound and then it was tightened and tied around the periosteal bridge and secured with polyester 5/0 suture.

**Figure 5:**
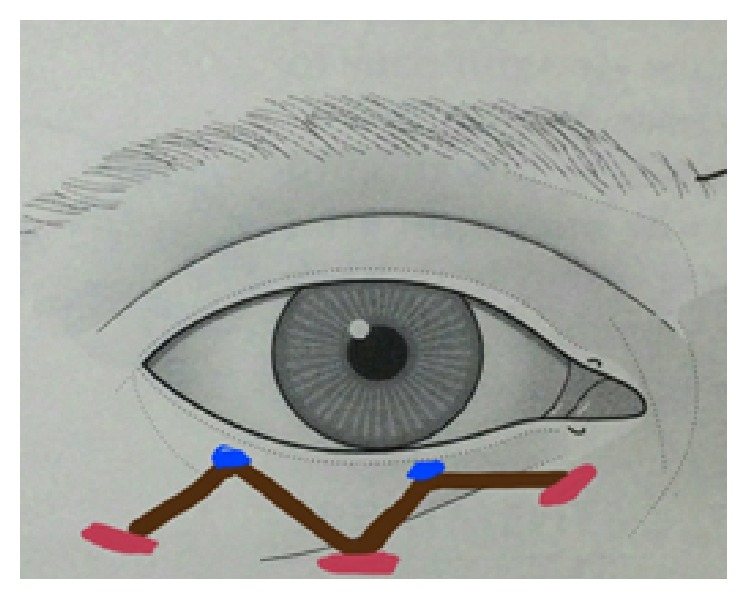
Drawing of the entire path of the fascia lata strip.

*In the Medial Cutaneous Incision*. The free end of the fascia strip was pulled to achieve the desired tension to adequately reform the inferior fornix and then the free end was sutured to the periosteum by 5/0 polyester suture (Figures 4 and 5).

The skin incisions were closed by 6/0 nylon sutures while the conjunctival incisions were left without sutures.

A proper sized conformer was placed to maintain the tissues in place for 3 weeks.

*(2) In Case of Contracted Socket*. A horizontal inferior fornical incision was performed and minimal dissection was done with excision of the fibrous bands if any to prepare the bed for the mucosal graft.

The buccal mucosal graft was sutured at the bulbar side of the conjunctiva and then the fascia lata strip was manipulated as described above and the palpebral side of the mucosal graft was sutured to the palpebral conjunctiva before tightening the fascia strip.

#### 2.3.3. Postoperatively

Medications included systemic broad spectrum antibiotic for one week and topical mixed antibiotic/steroid eye drops and ointment for 4 weeks.

**Figure 6:**
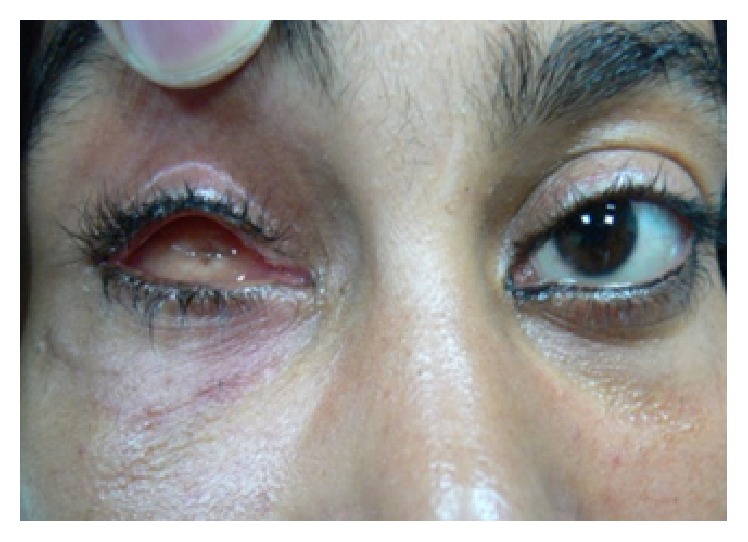
Postoperatively the inferior fornix was deep and the lower lid laxity was corrected.

**Figure 7:**
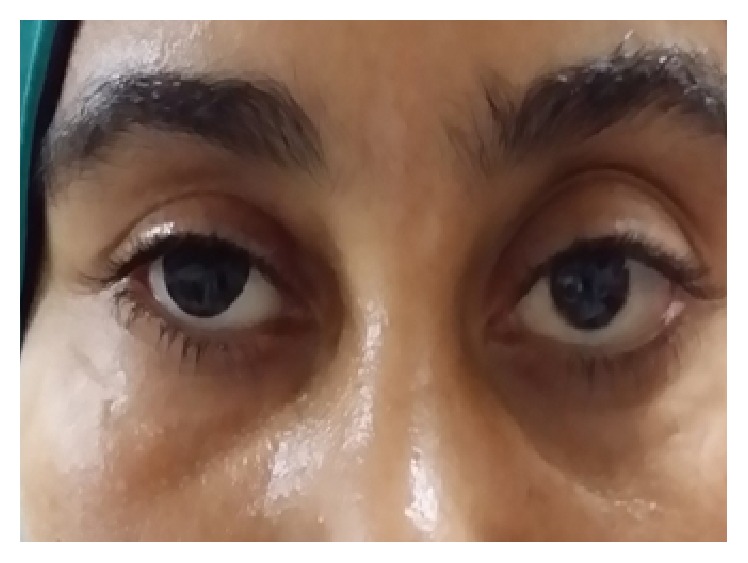
Postoperatively the patient can wear the ocular prosthesis comfortably.

Skin sutures were removed after one week and temporary conformer after 3 weeks (Figure 6) and then the patients wore their ocular prostheses (Figure 7).

One month postoperatively the central inferior fornix depth (CIFD) was measured in mm and recorded.

### 2.4. Statistical Analysis

After follow-up period, data were collected and analyzed using SPSS statistical program version 19 for windows and a statistically significant level was considered when *p* value was less than 0.05.

## 3. Results 

The follow-up period of all patients ranged from 9 to 26 months with a mean of 18.16 ± 4.56 months.

Successful procedure was considered if the patient could wear and retain the prosthesis in place comfortably in all circumstances without lower eyelid entropion, ectropion, or retraction (Figure 7).

## 4. Discussion 

The ultimate goal of socket surgery is to fit the patient with a prosthesis that would simulate as much as possible to his or her normal fellow eye [6].

The inability to retain an ocular prosthesis remains a psychologically devastating problem for patients [7] and a healthy and deep inferior fornix is particularly important factor in accommodating and supporting the ocular prosthesis [6].

Obliterated or shallow lower fornix might occur either in contracted socket with conjunctival scarring and foreshortening or in anophthalmic socket or postenucleation socket syndrome where there is abundant conjunctiva with lacked inferior fornix fixation [1].

Management of the shallow or obliterated lower fornix usually is directed towards the underlying aetiology.

In anophthalmic socket syndrome as there is no globe the inferior orbital fat migrated anteriorly and also the inferior rectus muscle is at a higher level in the socket with subsequent elevation of the lower lid retractors and their connections including the fornical conjunctiva. Prolapse of the fornical conjunctiva may result in anterior rotation of the lower edge of the prosthesis [3].

**Figure 8:**
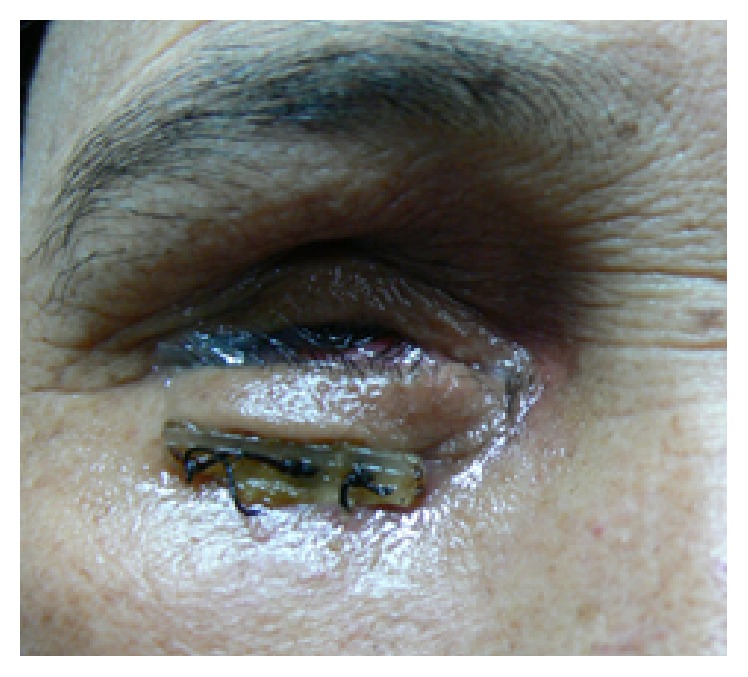
Deepening sutures tied over bolsters on the skin surface.

**Figure 9:**
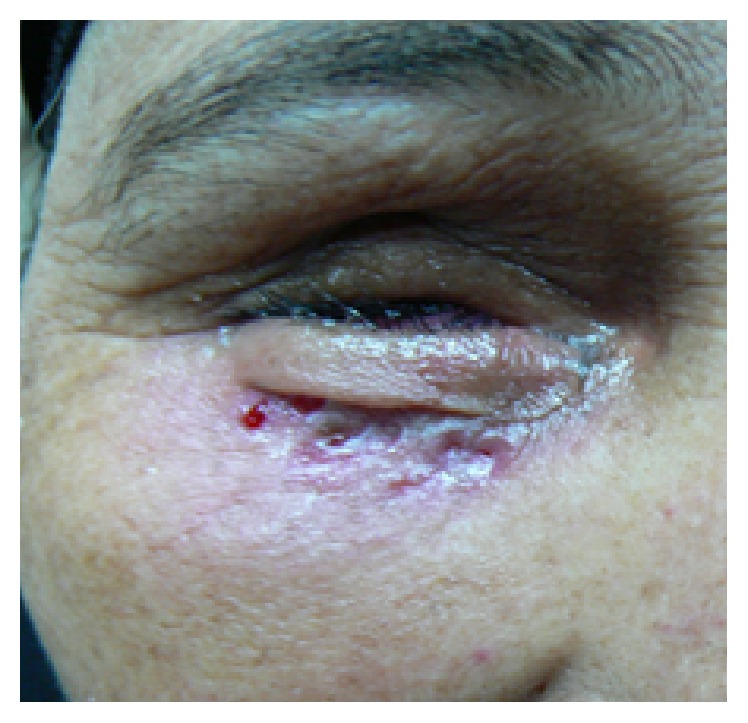
Early skin necrosis as a complication of deepening sutures.

In these cases, a silastic stent (e.g., 240 retinal band) can be positioned in the lowest aspect of the inferior fornix and anchored to the adjacent periosteum, with both arms of a double-armed suture passing through the stent, then through the deepest part of the fornix, through the periosteum of the inferior orbital rim, and through the full thickness of the eyelid. Silicone or rubber bolsters are used when the sutures are tied on the skin surface, anchoring the stent securely in the inferior fornix (Figure 8). These sutures may be removed in 2-3 weeks after adequate fibrosis has occurred between the inferior fornix and periosteum. A conformer must be in place all times until prosthesis is custom fitted [5]. But chronic mucoid conjunctival discharge and skin erosion with infection necessitated early removal of the externalized sutures and increased the risk of recurrence [4] (Figure 9).

Another method of repair consisted of a transconjunctival inferior fornix incision to gain direct exposure of the periosteum of the inferior orbital rim. Direct suture fixation of the edges of the conjunctival incision to the periosteum is then achieved. Externalized sutures and stents were not required [1, 8].

In contrary to anophthalmic socket syndrome where there is only poor lower fornix fixation, the contracted socket is characterized by inadequate conjunctiva in addition to cicatricial obliteration of the inferior fornix. So in management of contracted inferior fornix it is not enough to deepen the lower fornix but also additional tissue grafting is essentially needed.

Mucous membrane [2, 9] and amniotic membrane [10, 11] grafts are the most common tissue used in the reconstruction of the significantly contracted eye socket.

After the tissue graft has been sutured to the conjunctival edges, maintaining the graft in its bed and deepening of the inferior fornix can be achieved by various techniques including deepening sutures [3], slightly larger conformer with temporary tarsorrhaphy [12] or without tarsorrhaphy [13], or wire suturing of a custom conformer to the bone of the upper and lower orbital rim [14].

In the current study we used fascia lata strips to pull down and hold the lower eyelid retractors for deepening of the shallow inferior fornix in 24 cases. 9 patients had anophthalmic sockets and 15 patients had moderate to severe contracted sockets.

The patients of the study had undergone evisceration or enucleation for different reasons (Table 2) and with different periods since these procedures (Table 1).

Age of the patients ranged between 14 and 63 years with the mean age of 27.79 year (Table 1) and in comparison with the control group there was no statistically significant difference regarding the age (*p* = 0.3633).

41.7% of the patients were males while 58.3% of the patients had a left sided socket problem.

*Regarding the preoperative CIFD* in the patient group, the depth ranged from 0 to 4 mm with a mean of 1.875 mm (Table 1).

The mean preoperative CIFD was lower in anophthalmic subgroup (Table 3) and the difference between the 2 subgroups was statistically significant (*p* = 0.0342).

In the control group, the range was 6–11 mm with a mean of 8.375 mm (Table 1) and the difference between the control and the patient groups was statistically highly significant (*p* < 0.0001).

The postoperative CIFD after 1 month in all patients was markedly increased and it ranged from 3 to 9 mm with an average of 6.833 mm (Table 3) and these changes were statistically highly significant when compared to the preoperative CIFD (*p* < 0.0001).

The improvement in mean postoperative CIFD was higher in anophthalmic than in contracted subgroup (Table 3) and the difference between the 2 subgroups was significant (*p* = 0.0028).

After a follow-up of average 18 months, 95.8% (23 out of 24) of patients had satisfactory results as they could wear and retain their prostheses all the time without significant problems.

100% of anophthalmic socket patients and 93.3% of cases with contracted sockets achieved satisfactory postoperative results during the follow-up period (Table 2).

Regarding the success rate, there was no significant statistical difference in both anophthalmic and contracted socket subgroups (*p* = 0.4).

16 cases (66.6%) needed reconsultation of an ocularist for larger prosthesis to match the newly formed fornix and we did not encounter any case of lower eyelid entropion, ectropion, retraction, or lagophthalmos during the follow-up period.

The only case that failed to retain the prosthesis was a 23-year-old female with severe left contracted socket and later on she required another 2 socket operations for buccal mucosal graft and finally the result was satisfactory for her.

The 9 cases with anophthalmic socket syndrome had preoperative lax lower lids (Figure 1) which were spontaneously corrected after the fascia lata technique without need for lateral tarsal strip (Figure 6).

6 out of 15 cases (40%) with contracted socket had shallow superior conjunctival fornix and were managed simultaneously with buccal mucosal grafts that held in place with the conformer.

All cases in our study reported pain at the lower outer side of the thigh for only one week and only 5 cases (21%) reported limbing during the same period.

None of the cases reported ugly scar, haematoma, or muscle herniation; this might be due to the small skin incision (2 cm) and narrow fascia lata harvested (3 mm).

In comparison to deepening sutures, fascia lata is autogenous and it is buried in the tissues so no skin necrosis or infection was anticipated. Also fascia lata technique is not a temporary procedure and so the desired pulling down tension on the lower lid retractors is expected to be maintained for long time as the nonelastic fascia is now fixed to the tough nonresilient periosteum.

Moreover intraoperatively the tension exerted by the fascia on the lower lid retractors can be judged and monitored before tying and suturing the medial end of the fascia strip and so postoperative lower lid entropion or retraction can be avoided.

In the conjunctival fixation technique described by Neuhaus and Hawes [1] and modified by Ma'luf [8], good results were reported in all of the 12 patients of the study. All of the patients became able to retain a prosthetic eye postoperatively with two patients having minimal postoperative lower eyelid retraction and another two patients having minimal lower eyelid entropion but no secondary surgery was required.

In spite of the above good results of the conjunctival fixation technique, it seems that the fascia lata technique is superior for the following reasons:

1. The conjunctival fixation is a technique that cannot be used in contracted socket due to the lack of sufficient conjunctiva to be fixed to the periosteum in these cases.
2. Fascia lata technique is more physiologic than conjunctival fixation to the periosteum as the former aimed at lowering and pulling down the abnormally elevated lower lid retractor to a near normal position and not to divide the lower lid retractors to secure the conjunctival edge to the periosteum.
3. In anophthalmic socket, it is preferable not to manipulate or insult the precious conjunctiva too much to avoid triggering cicatrisation and the development of contracted socket. In fascia lata technique, the conjunctiva was minimally manipulated in contrary to the long conjunctival incision and deep tissue dissection in conjunctival fixation procedure.

The drawback of our technique is that it should be performed under general anesthesia. In addition, harvesting fascia lata is not a simple procedure and it is time consuming with a second operation site in the thigh with its complications.

## 5. Conclusion 

The fascia lata technique is a new, alternative, and effective procedure to correct the shallow inferior fornix in anophthalmic socket syndrome where minimal socket dissection is required and complications of the deepening sutures are to be avoided also in moderate to severe contracted sockets fascia lata technique which is a second alternative to deepening sutures but avoids their complications.

## Figures and Tables

**Table 1:** Depicting demographic data and preoperative CIFD of control and patient groups.

Variable	Control group	Patient group
Number	*24*	*24*

*Age (years)*		
Range	10–67 years	14–63 years
Mean and standard deviation	31.92 ± 14.63	27.79 ± 16.48

*Sex *		
Male	9 (37.5%)	10 (41.7%)
Female	15 (62.5%)	14 (58.3%)

*Side of the socket problem*		
Right	11 (45.8%)	10 (41.7%)
Left	13 (54.2%)	14 (58.3%)

*Time elapsed since evisceration or enucleation in years *		
Range	1–9 years	3–16 years
Mean and standard deviation	5.083 ± 2.020	5.833 ± 2.697

*Preoperative CIFD in mm*		
Range	6–11 mm	0–4 mm
Mean and standard deviation	8.375 ± 1.469	1.875 ± 1.191

**Table 2:** Depicting indications for evisceration and enucleation in patient group.

Indication for evisceration or enucleation	Number and percentage
(1) *Blind painful eye* (neovascular glaucoma, perforated corneal ulcer, and endophthalmitis)	9 (38%)
(2) *Disfiguring eye* (atrophia or marked anterior staphyloma)	8 (33%)
(3) *Trauma*	7 (29%)

**Table 3:** Preoperative and postoperative CIFD and success rate in anophthalmic and contracted subgroups and in all patients group.

	Anophthalmic subgroup	Contracted subgroup	All patients group
*Preoperative CIFD in mm*			
Range	0–3 mm	0–4 mm	0–4 mm
Mean and standard deviation	1.222 ± 1.202	2.267 ± 1.033	1.875 ± 1.191

*Postoperative CIFD in mm*			
Range	7–9 mm	3–8 mm	3–9 mm
Mean and standard deviation	7.889 ± 0.7817	6.200 ± 1.373	6.833 ± 1.435

*Success rate*	100%	93.3%	95.8%
